# Elevation in Cell Cycle and Protein Metabolism Gene Transcription in Inactive Colonic Tissue From Icelandic Patients With Ulcerative Colitis

**DOI:** 10.1093/ibd/izy350

**Published:** 2018-11-19

**Authors:** Mathena Vinayaga-Pavan, Matthew Frampton, Nikolas Pontikos, Adam P Levine, Phillip J Smith, Jon G Jonasson, Einar S Björnsson, Anthony W Segal, Andrew M Smith

**Affiliations:** 1 Microbial Diseases, Eastman Dental Institute; 2 Molecular Medicine, Division of Medicine; 3 UCL Genetics Institute, University College London, London, United Kingdom; 4 Department of Pathology, Landspitali University Hospital Reykjavik, Iceland; 5 Faculty of Medicine, University of Iceland, Reykjavik, Iceland; 6 Department of Gastroenterology and Hepatology, Landspitali University Hospital, Reykjavik, Iceland

**Keywords:** microarray, inflammatory bowel disease, rectum, TPMT

## Abstract

**Background:**

A combination of genetic and environmental factors is thought to be involved in the pathogenesis of ulcerative colitis (UC). In Iceland, the incidence of UC is one of the highest in the world. The aim of this study was to characterize patients with UC and identify potential germline mutations and pathways that could be associated with UC in this population.

**Methods:**

Exome sequencing and genome-wide microarray analysis on macroscopically noninflamed colonic mucosa from patients and controls were performed. Exome sequence data were examined for very rare or novel mutations that were over-represented in the UC cohort. Combined matching of variant analysis and downstream influence on transcriptomic expression in the rectum were analyzed.

**Results:**

One thousand eight hundred thirty-eight genes were differentially expressed in rectal tissue from UC patients and identified an upregulation in genes associated with cell cycle control and protein processing in the endoplasmic reticulum (ER). Two missense mutations in thiopurine S-methyltransferase (TPMT) with a minor allele frequency of 0.22 in the UC patients compared with a reported 0.062 in the Icelandic population were identified. A predicted damaging mutation in the gene SLC26A3 is potentially associated with increased expression of DUOX2 and DUOXA2 in rectal tissue.

**Conclusions:**

Colonic mucosa of UC patients demonstrates evidence of an elevation in genes involving cell proliferation and processing of proteins within the ER. Exome sequencing identified a possible increased prevalence of 2 damaging TPMT variants within the UC population, suggesting screening the UC population before initiation of thiopurine analogue therapy to avoid toxicity associated with these mutations.

## INTRODUCTION

Ulcerative colitis (UC) is a chronic immune-mediated condition primarily affecting the colonic epithelium. The pursuit of the genetic contribution to this disease has, in recent years, focused on several large-scale genome-wide association studies (GWAS), with >240 risk loci for inflammatory bowel disease (IBD) identified to date.^1^ Of the 240 loci identified, only 28 are specific to UC, with 141 being also being associated with Crohn’s disease.^2^ Indeed, estimates of the genetic correlation suggest only a small influence on disease variance.^3^ The reduction of costs in technology facilitating assessment of larger quantities of genetic data allows multi-omic data to be used to investigate missing heritability and possible environmental factors contributing to disease. The transcriptomic alterations within the inflamed colonic mucosa in IBD have been well documented, and a meta-analysis conducted by Clark et al. combined 6 independent microarray data sets and performed a comprehensive bioinformatic analysis.^4^ Consistent with genome-wide association study (GWAS) data, cellular processes relating to immune defense response, leukocyte activation, cell proliferation, and response to bacterial components were identified. Previous studies on noninflamed colonic mucosa from UC patients have identified a number of potential causally related genes such as bone morphogenetic protein/retinoic acid–inducible neural specific 3 (BRINP3),^5^ but all of these studies have been conducted on patients with varying degrees of underlying microscopic inflammation and a mixed ethnicity that was not genetically verified. This heterogeneity may impact on the ability to identify subtle but relevant changes to the mucosal tissue.

Ulcerative colitis has an incidence of 16.5 per 100,000 in the population of Iceland, equating to one of the highest incidence in the world.^6^ The most recent nationwide population-based study of the incidence of IBD in Iceland between 1995 and 2009 identified a significant increase in UC over the study period, whereas Crohn’s disease remained stable, with a much lower incidence of 6.6 per 100,000.^7^ The population of Iceland is approximately 340,000,^8^ and genetic studies have revealed a reduced genetic heterogeneity, with genetic drift having a greater impact on the genetic variation when compared with other European countries due to geographic, and hence reproductive, isolation.^9^ A previous study into the genetic contribution to IBD in the Icelandic population identified a high degree of familial aggregation that extended beyond the nuclear family.^10^ These findings suggest that a genetic predisposition in combination with a potential environmental effect is likely to be driving the increased prevalence of UC in this population.

This study aims to combine exome sequencing of genomic DNA and transcriptomic data from noninflamed colonic mucosal tissue collected from well characterized Icelandic UC patients and ethnically matched healthy controls. The data will be used to identify novel genetic variants and transcriptomic signatures that will improve our understanding of the etiopathogenesis of the disease and potentially highlight new therapeutic targets.

## METHODS

### Patient Recruitment, Ethics, and Tissue Collection

All patients were recruited from the Gastroenterology and Hepatology Department at the Landspitali University Hospital Hringbraut, Reykjavik, Iceland. Ulcerative colitis patients had histologically confirmed disease before recruitment. Consent was obtained under approval from the Bioethics Committee of Iceland (application number VSNb2011080005/03.15) and the Data Protection Authority of Iceland. Healthy controls (HCs) were recruited from individuals undergoing colonoscopy, matched for sex and age (±5 years) (Table 1). Biopsies were collected during routine colonoscopies and were performed on UC patients undergoing surveillance. All patients had clinically quiescent disease for a minimum of 3 months before the procedure and upon inspection had a MAYO endoscopic score assessed as ≤1.^11^ Paired endoscopic pinch biopsies (Olympus biopsy-forceps, FB-24U-1) were obtained from the ascending colon and rectum. One biopsy was placed in RNAlater stabilization reagent (Qiagen, Hilden, Germany) and stored at −80°C for messenger RNA (mRNA) preparation, and the other was placed in 4% formaldehyde for histological evaluation using the Geboes scoring system.^12^ Peripheral venous blood samples were taken from all study participants at the time of endoscopy for the assessment of systemic inflammatory status and to obtain genomic DNA.

**Table 1.** Characteristics of Subjects

	Ulcerative Colitis		Healthy Controls
No.	18		17
Age (range), y	49 (29–77)		46 (19–75)
Sex			
Male	7		6
Female	11		11
Extent of disease (E1/E2/E3)	Extent at index colonoscopy	Extent at 5-y follow-up	—
	E1 = 5	E1 = 5	
	E2 = 8	E2 = 7	
	E3 = 4	E3 = 5	
Medication, No. (%)			—
No treatment	3 (17)	4 (22)	
5-ASA	15 (83)	10 (56)	
Azathioprine		3 (17)	
Biological therapy		1 (5)	
Smoking status, No. (%)			
Current smoker	3 (17)		3 (17)
Ex-smoker	7 (39)		5 (29)
Never smoked	8 (44)		9 (54)
Microscopic inflammation within the rectum Geboes histological score	0 = 8		0 = 14
	1.1 = 4 1.2 = 1		


There was no significant difference in age and sex. Extent of disease is based on Montreal classification. Microscopic inflammatory status of the rectum was verified by histopathology assessment using Geboes scoring (0 = no abnormality; 1.1 = mild but unequivocal increase in chronic inflammatory infiltrate; 1.2 = moderate increase in chronic inflammatory infiltrate).

Abbreviations: 5-ASA, aminosalicylate; E1, proctitis; E2, left-sided colitis; E3, pancolitis.

### RNA Extraction and Genome-Wide Transcriptomic Analysis

Sixty-seven biopsies (∼25 mg/biopsy) were lysed in 300 μL of RNeasy Fibrous Tissue kit (Qiagen) RLT buffer (Qiagen) and 0.14 M of β-mercaptoethanol (β-ME; Sigma-Aldrich, St Louis, MO, USA) and then homogenized by centrifugation at 10,000*g* through a Qiashredder column (Qiagen). Protein was removed by incubation for 10 minutes at 55°C with 10 μL of Proteinase K (20 mg/mL; >600 mAU/mL; Qiagen). Total RNA was extracted on RNeasy Mini-Spin Columns, and DNA was removed with RNase-Free DNase Digestion (Qiagen). Samples were taken forward for further assessment if there was an RNA concentration of >25 ng/μL using the Qubit 2.0 fluorometer. All samples analyzed were optimized with ethanol precipitation to achieve an RNA integrity number of >7.0 using an Aligent 2100 bioanalyzer or optical density ratio of >1.8 OD_260_/OD_280_ and >1.8 OD_260_/OD_230._

For each biopsy sample, 250 ng of total RNA was amplified and purified using the Illumina TotalPrep-96 RNA amplification kit (Ambion; Life Technologies, Carlsbad, CA, USA). Biotin-labeled complementary RNA (750 ng at 150 ng/μL) was hybridized to Illumina HumanHT-12v4 Expression BeadChips (Illumina, San Diego, CA, USA) for 16 hours at 58°C. After hybridization, BeadChips were washed and stained with streptavidin-Cy3 (GE Healthcare, Little Chalfont, UK), scanned using the BeadArray reader (Illumina), and processed using Illumina Genome Studio software.

### Bioinformatic Analysis of the Genome-Wide Transcriptomic Data

For each individual, our multi-omic data set contains both exome sequencing data and transcriptomic profile data from 1 or more biopsies from the ascending colon and/or rectum. Allele-sharing distance analysis revealed 1 duplicate exome, and so after removing the corresponding individual from the data set, 35 remained: 18 cases and 17 controls. Analyses were performed using Python 2.7 and R, version 3.3. The FactoMineR R package^13^ was used for principal component analyses (PCAs).

### Exome Sequencing Data Analysis

Indexed paired-end libraries were prepared using the BGI 59 Exome Enrichment Kit, and 2×150 base pair (bp) sequencing was performed by BGI on the Illumina HiSeq 2000 system. Read alignment and variant calling of the exomes were performed simultaneously with ~5K other exomes using an in-house next-generation sequencing analysis pipeline.^14^ Reads were aligned to the human reference genome using hg37 by Novoalign (version 3.02.08), and variants were called by the Genome Analysis Toolkit (GATK) according to best practices, that is, local realignment around InDels, followed by joint variant calling and variant quality score recalibration.^15^ Further requirements for quality assurance included:

1. genotyping quality (GQ) ≥20;
2. reference/alternate read depth for heterozygote calls does not diverge significantly from 50:50 (chi-square test *P* ≥ 0.001);
3. alternate read depth for heterozygote and homozygote alternate calls ≥3;
4. GATK Variant Quality Score Recalibration (VQSR) truth tranche ≤99.5% for single nucleotide polymorphsims (SNPs) and ≤99.0% for InDels;
5. no-call rate in our samples ≤0.25;
6. genotypes do not diverge significantly from the Hardy-Weinberg Equilibrium (chi-square test, *P* ≥ 5×10^–8^).

Variants were annotated by the Variant Effect Predictor (VEP; version 76).^16^

An ancestry PCA was performed on the Icelandic exomes (ICE) to confirm their ancestry as European and homogeneous. Around 5000 independent common SNPs (minor allele frequency [MAF] ≥ 5%) from across the exome were used, plus 1000 Genomes Project samples from European, East Asian, and African populations.^16^ In addition, the allele-sharing distance (ASD) in these SNPs was calculated for each sample pair within the ICE, British in England and Scotland (GBR), and Toscani in Italy (TSI) populations, and then 1-way ANOVAs were used to indicate whether (1) Icelandic samples are relatively genetically homogeneous compared with other European subpopulations; (2) a difference exists in the within- vs between-group relatedness of UCs and HCs. Note that for (1), no firm conclusions can be drawn because the sample size is small and the GBR and TSI samples were sequenced separately (by the 1000 Genomes Project). If (2) showed a difference, then it would be a potential source of bias in downstream analyses.

Potentially damaging variants were identified by the following filters:

1. frameshift, stop gain, splice site changes, and missense variants predicted as damaging by 1 or more of the following: SIFT, PolyPhen-2,^17^ Condel,^18^ CAROL,^19^ Combined Annotation Dependent Depletion (CADD)^20^ algorithms. Standardized cutoff for pathogenicity prediction cited by the authors was used to identify potentially damaging variants. For PolyPhen-2 predictions, the “probably damaging” and “possibly damaging” categories were considered damaging.
2. minor allele frequency (MAF) <0.05 in Kaviar^21^ and in non-Finnish Europeans in the Exome Aggregation Consortium (ExAC)^22^ database and Genome Aggregation Database (gnomAD).
3. present in at least 25% of our cases.

Each variant was tested for association with UC vs the sequenced healthy controls and using previously published Icelandic allele frequencies using the Fisher exact test.^23^

### Transcriptome Expression Microarray Analysis

The original expression data contained 47,198 probes and 57 samples: 27 ascending colon and 30 rectal taken from the 35 individuals. For ascending colon samples, there were 12 cases and 15 controls, and for rectal samples, 15 cases and 15 controls.

These data were log_2_-transformed, quantile-normalized, and analyzed in R using various Bioconductor packages.^24, 25^

Probes with a minimum detection *P* value of <0.01 in at least 2 biopsies were retained (n = 22,716). Two case samples (1 ascending colon, 1 rectal) with significantly lower numbers of detected probes were excluded. Retained probes were mapped to gene IDs by the illuminaHumanv4.db package. Adjustment of the expression data for chip-level batch effects was done by the empirical Bayes method ComBat^26^ (sva R package).^27^ The recorded bowel sites were verified by examining expression of genes known to significantly differ between the ascending colon and rectum and through a PCA on all probes (data not shown): 1 ascending colon and 2 rectal samples demonstrated discordant expression from what was expected and were removed. Post-quality control (QC), there were 52 samples (24 cases, 28 controls), of which 25 were ascending colon (11 cases, 14 controls) and 27 were rectal (13 cases, 14 controls).

Differential expression analysis was performed with the LIMMA package.^28^ For each bowel location, samples were grouped by disease status (case vs control or Montreal classification^29^), and *P* values were adjusted for multiple testing by the Benjamini-Hochberg procedure (adj *P* value).^30^ Ancestry was controlled for by using coordinates from the exome data PCA as covariates.

For gene ontology (GO) and Kyoto Encyclopedia of Genes and Genomes (KEGG) analysis, STRING^31^ was employed. Where a transcript had 2 probes or more, the probe with the highest fold change or expression values was used for analysis. Pathway analyses were also conducted using DAVID Bioinformatics Resources 6.7^32, 33^ and WebGestalt^34^ to ensure consistency of results.

### Determination of Gene Expression Influence by Predicted Damaging Variants

Differential expression analysis was performed with the LIMMA package. For each damaging variant, samples were grouped by wild-type or carrier of the variant, and *P* values were adjusted for multiple testing by the Benjamini-Hochberg procedure.

### Integrated Multi-omic and Clinical Data Analysis

Differential expression analysis was also performed where samples were grouped by genotype for prioritized potentially damaging coding variants. A further PCA was performed on the multi-omic and clinical data together. The latter included sex, microscopic inflammation score, disease status, disease extent (Montreal classification for each patient), treatment status, hemoglobin, neutrophil count, lymphocyte count, albumin (ALB), C-reactive protein (CRP), alkaline phosphatase (ALP), gamma glutamyl transferase (ɣGT), aspartate transaminase (AST), alanine transaminase (ALT), and bilirubin. The aim was to assess the relative utility of these various data.

## RESULTS

### Assessment of Ancestry and Relatedness

**FIGURE 1.**
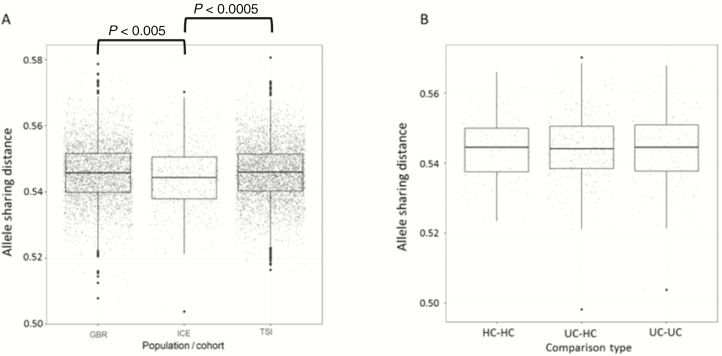
Box plots of pairwise ASD across the exome in sample groups. A, 1000 Genomes Project GBR population, all samples in this study (ICE), 1000 Genomes Project TSI population. B, HC, UC patients, and UC-HC, UC.

In the ancestry PCA, the Icelandic samples (ICE) clustered together in close proximity to the 1000 Genomes Project GBR and Utah Residents (CEPH) with Northern and Western Ancestry samples (Supplementary Fig. 1), confirming their ancestry as European and homogeneous. In the assessment of relatedness, there was a difference (*P* < 0.0005) in the mean ASD of GBR, TSI, and ICE samples (Fig. 1A). According to Tukey’s honest significant difference method, there was a significant difference between ICE and both GBR and TSI (*P* < 0.005 and *P* < 0.0005), but not between GBR and TSI. In line with other studies, this suggests that the Icelandic population is relatively genetically homogenous, but as stated in the “Methods,” no firm conclusions can be drawn. There was no significant difference in the mean ASD of HC, UC-HC, and UC (Fig. 1B), thus providing confidence that downstream analyses are not confounded by a difference in the within- vs between-group relatedness of UCs and HCs.

### Identification of Rare and Damaging Mutations in the Icelandic UC Cohort

Exome sequencing resulted in the identification of 12,483 predicted damaging variants within the entire cohort (n = 35). Further filtering based on our bespoke filtering (see the “Methods”) reduced this to 238; of these variants, 48 had a CADD score greater than 20, and 38 were deemed to have a high impact on gene function based on VEP consequence descriptors (Supplementary Table 1).^35^ Where available, comparisons were also made with the Decode Icelandic whole-genome database (n = 2363), available from the European Variant Archive (PRJEB8636).^23^ It was possible to test for enrichment in our cohort compared with the Icelandic population for 50 of the 252 variants within our total cohort. We identified 2 damaging missense mutations in thiopurine S-methytransferase (TPMT), A154T/rs1800460, and Y240C/rs1142345, in 8 out of 18 UC patients (44%), and 2 out of 17 (12%) healthy controls (Supplementary Table 2). All 8 patients were found to be heterozygous, with an MAF of 0.22 for both variants compared, with the reported Icelandic population frequency of 0.062 and 0.063, respectively (Decode data set). Overall the Icelandic population has a higher MAF for both variants in TMPT compared with the non-Finnish European population (MAF, 0.03 and 0.04, respectively). These 2 variants are in ~100% linkage disequilibrium (LD) in the Icelandic population and are known to result in a loss of enzyme function leading to high levels of toxic metabolites from thiopurine analogues, which cause liver toxicity and bone marrow suppression.^36, 37^

### Predicted Damaging Variants in Genes Identified by Previous GWAS Studies

To date, 240 loci have been identified associated with IBD by GWAS.^1^ Of the 248 predicted damaging variants identified as enriched within the Icelandic UC patients, 7 were located within IBD-associated loci. These included *SLC26A3*, *GPR35*, *CD226*, *F5*, *SKAP2*, *INFG*, *NXPE1*, and *SLC39A11*. Only the variants in *SLC26A3* and *F5* had a CADD score above 20, and none of these reached significance after correction for multiple testing.

### Characteristics and Inflammatory Status of UC Patients

**FIGURE 2.**
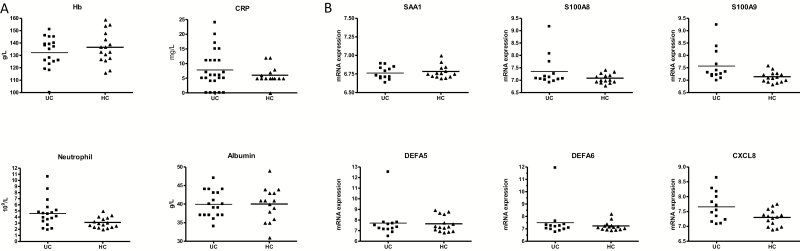
Assessment of inflammatory status of UC patients and controls. A, Systemic inflammatory markers from peripheral blood samples taken from subjects on the day of endoscopy (CRP, Hb). B, Rectal biopsy transcriptomic profiles of known inflammatory genes for all UC and HC subjects (SAA1, serum amyloid 1; S100A8, S100 calcium binding protein A8; S100A9, S100 calcium binding protein A9; DEFA5, defensin A5; DEFA6, defensin A6; CXCL8, interleukin-8). One UC patient was identified to have an elevated inflammatory transcriptomic signature.

Summary characteristics of the study cohort are recorded in Table 1. The serum levels of inflammatory markers CRP and albumin, in addition to circulating peripheral neutrophil numbers, were not significantly different between the HC and UC cohorts (Fig. 2A). In addition, all other parameters tested were similar between the HC and UC (see the “Methods,” data not shown). A PCA of the entire blood results demonstrated no difference between the HC and UC groups (data not shown). MAYO scoring of all patients confirmed the quiescent nature of the disease activity, with 5 patients having a MAYO score of 1 identified in the rectum and all other patients scoring 0. Histological assessment of the rectal and ascending colonic biopsies revealed microscopic inflammatory activity in 5 of the UC biopsy samples from the rectum and none in the ascending colon. The microscopic activity within the rectum was 1.1 in 4 patients and 1.2 in 1 UC patient, with all other biopsies scoring 0 using the Geboes scoring system. PCA did not separate this cohort from the microscopically noninflamed UC biopsies. None of the HC biopsies were found to have microscopic inflammation or any other pathological abnormalities. On review of the medical records for the control subjects 5 years after the biopsies were collected, none had developed IBD or any other colonic pathology.

### Identification of Different Inflammatory States Within the Rectum of UC Patients Using Transcriptomics

Previous studies conducted on inflamed biopsy material from UC patients by Noble et al. identified an inflammatory gene signature.^38^ The major inflammatory markers were reported to be SAA1, S100A8, S100A9, DEFA5, DEFA6, and IL-8. We examined the expression of these genes in our Icelandic cohort to ascertain the inflammatory status of the rectum and ascending colon at the transcriptomic level (Fig. 2B). One individual demonstrated an elevation in DEFA5 and DEFA6 (Fig. 2B). Three of 6 individuals with microscopic inflammation had elevated levels of S100A8 and S100A9 when compared with the average of the cohorts. The remaining 9 UC patients and all of the HCs (n = 15) were found to have similarly low levels of all 6 inflammatory markers. Overall there was no significant group difference in the expression of the classical inflammatory genes between the UC and HC groups.

### Change in Global Transcriptomic Profile in UC Rectum

**FIGURE 3.**
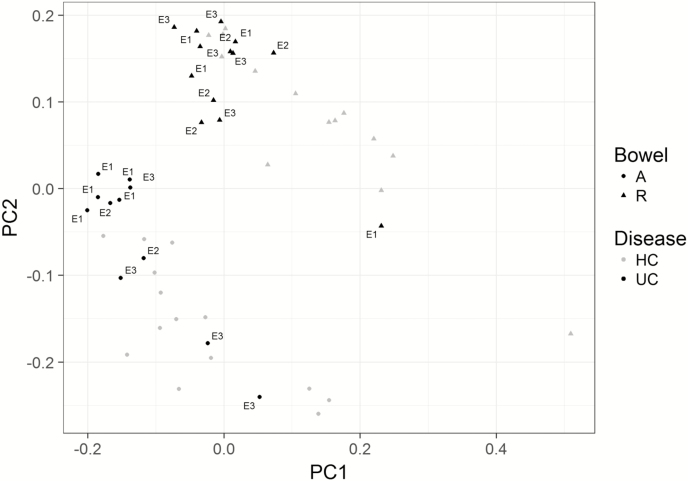
PCA of microarray transcriptome data from bowel biopsies: The biopsies are distinguished by location (ascending colon [circle] or rectum [triangle]), disease status (UC [black] or HC [gray]), and Montreal classification of the UC (E1, proctitis; E2, left-sided colitis; E3, pancolitis).

**FIGURE 4.**
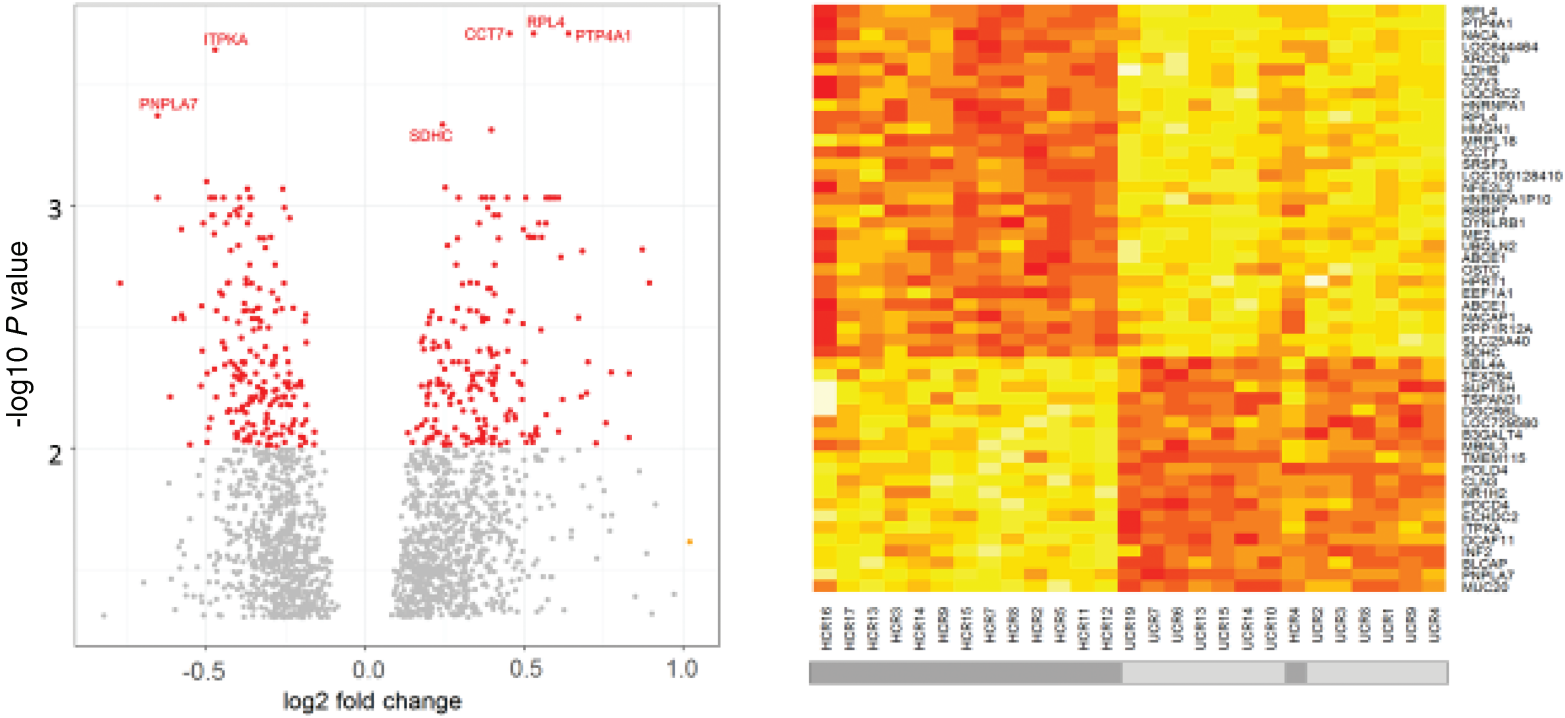
Differential gene analysis of noninflamed rectal biopsies from UC patients and HCs. A, Volcano plot of all HC vs UC rectal genes. Genes colored red are significantly different, with an adjusted *P* < 0.01. B, Heatmap of the 50 most differentially expressed genes between UCs and HCs (according to adj *P* value), where the color scale for relative expression level is from low (red) to high (white).

Principal component analysis and differential gene analysis were performed on the entire UC (n = 18) and HC (n = 16) transcriptomic data sets (n = 22,716 probes). Principal component analysis separated the samples by site of biopsy and disease status (Fig. 3). Differential analysis comparing rectal gene expression in UC and HC subjects identified 1838 differentially expressed genes, of which 935 genes were overexpressed and 903 underexpressed in the disease cohort (adj *P* < 0.05) (Fig. 4; Supplementary Table 3).

The GO and KEGG pathway analysis of the upregulated probes (n = 935) within the rectal tissue of Icelandic UC patients revealed an enrichment for ribosomes (KEGG pathway 03010; false discovery rate, 0.00098) and protein processing in the endoplasmic reticulum (KEGG pathway 04141; false discovery rate, 0.00628) (Table 2).^39–42^ Analysis of the biological process and molecular process of gene ontology identified 2 groupings that have been previously established as important in the pathogenesis of UC, including unfolded protein response (UPR)^43^ and macroautophagy^44^ (Supplementary Fig. 2).

**Table 2.** Gene Ontology and KEGG Pathway Analysis of Differential Genes From the Rectum

Pathway ID	Pathway Description	Count in Gene Set	False Discovery Rate
KEGG Pathway			
03010	Ribosome	13	0.000982
04141	Protein processing in endoplasmic reticulum	13	0.00628
05016	Huntington’s disease	12	0.0483
Biological process (GO)			
GO:0044237	Cellular metabolic process	237	1.65e-11
GO:0043170	Macromolecule metabolic process	214	3.23e-11
GO:0044260	Cellular macromolecule metabolic process	202	3.23e-11
GO:0071704	Organic substance metabolic process	233	9.17e-09
GO:0044238	Primary metabolic process	228	1.45e-08
Molecular process (GO)			
GO:0003723	RNA binding	78	7.68e-11
GO:0044822	Poly(A) RNA binding	66	7.68e-11
GO:0003676	Nucleic acid binding	121	1.99e-05
GO:1901363	Heterocyclic compound binding	159	4.14e-05
GO:0097159	Organic cyclic compound binding	160	4.67e-05

Examination of the upregulated rectal genes (n = 935) within the Icelandic UC patients revealed an enrichment for ribosomes and protein processing in the endoplasmic reticulum.

### Ascending Colon Transcriptomic Data

Differential analysis identified 184 differentially expressed gene probes between ascending colonic tissue from the UC and HC cohorts. Sixty-three genes were upregulated, and 121 genes were downregulated. No GO or KEGG pathways were significantly enriched, and STRING analysis was not able to identify any significant groupings.

The confluent distal to proximal colonic inflammation characteristic of UC provides the opportunity to assess areas of the bowel with repeated episodes of inflammation with more proximal areas that were likely to have a reduced burden of inflammation or no inflammation at all. To identify if there was evidence of an intrinsic transcriptomic signature that was independent of inflammation, we compared the differential transcriptomic analysis of the ascending and rectal biopsies. Thirty-seven gene probes were identified as overlapping in both groups of transcriptomic data (Supplementary Table 4). Fifteen genes were underexpressed and 22 overexpressed, and all but 1 gene had the same directional fold change in both areas of the bowel. The genes identified primarily relate to cell cycle, ribosomes, transcription factors, and cytoskeleton formation. There was no significant GO term enrichment for these groups of genes.

### Potential Impact of the Rare Damaging Variants on Rectal Gene Expression

**FIGURE 5.**
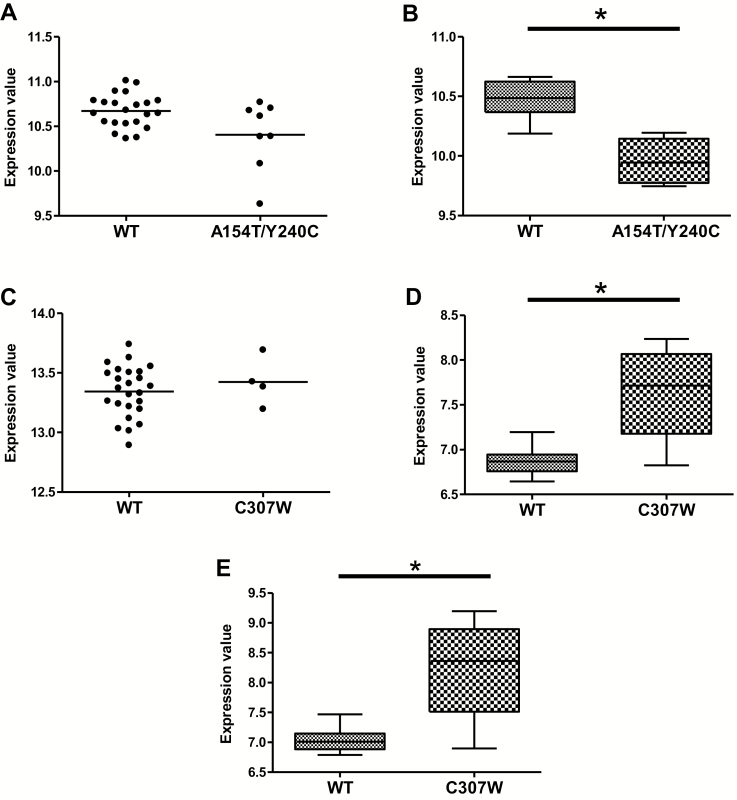
Variant impact on downstream transcriptomics. A, Rectal expression of TPMT is not affected by the 2 observed TPMT variants (A154T/Y240C), (B) whereas the variants demonstrate a significant downstream impact on SPOP expression within the rectum. C, Expression of SLC26A3 within the colon is not affected by variant status (C307W), but (D) and (E) carriers of this mutation demonstrate an increase in DUOXA2 and DUOX2 expression within the rectum. *Adj *P* < 0.05.

There was no significant difference in the burden of damaging variants when comparing the UC group and the HC group (data not shown). Through a combined analysis of exome variants and transcriptomic data, we were able to determine if rare predicted damaging variants observed within the UC cohort had any influence on rectal gene expression. Within the group of 248 prioritized variants, several were found to have a potential influence on rectal and ascending colon gene expression. Individuals that carry the 2 TPMT mutations had a decreased expression of speckle type BTB/POZ protein (SPOP; adj *P* < 0.05) (Fig. 5B). Carrying the mutation C307W in the *SLC26A3* gene was associated with an increase in expression of both the dual oxidase 2 (DUOX2) and dual oxidase maturation factor 2 (DUOXA2; adj *P* < 0.05) (Fig. 5D and E).

## DISCUSSION

Ulcerative colitis is a complex disease with both genetic and environmental factors contributing to its pathogenesis. Previous genetic studies have identified large numbers of common genetic variants that together are thought contribute to an increased risk of developing UC via modulation of pathways and processes. To identify the pathways and processes that are abnormal in UC, it is preferable to study a population with a high disease incidence that is ethnically homogeneous and from a similar environment. The Icelandic population fulfills these requirements. By studying exome and transcriptomic data from Icelandic UC patients, we have provided evidence to support an alteration in colonic mucosal homeostasis and the upregulation in pathways of protein processing within the endoplasmic reticulum.

Genomic data from the Icelandic cohort under study have reconfirmed the narrowed heterogeneity described by Helgason et al.^9^ showing a nonrelated but genetically restricted population. To identify genes and processes that may be implicated in the disease etiology separate from the transcriptomic signature of inflammation, we specifically targeted patients with quiescent disease. The analysis of the histology, systemic inflammatory markers, and whole-genome transcriptomic profiling of our subjects demonstrated that the current cohort represents a clinically quiescent group of patients. These results provided us with confidence that our findings were not directly associated with an ongoing chronic inflammatory process or infiltrating leukocyte populations, but were associated with changes within the cells of the colonic mucosa. Our results demonstrated some overlap with a number of previous transcriptomic studies conducted in different ethnic populations.^5, 45^ We have previously conducted a transcriptomic analysis on macroscopically normal bowel pinch biopsies obtained from patients in the United Kingdom and identified 3 abnormally expressed genes in UC rectal tissues.^5^ The Icelandic UC biopsies had a significantly higher number of differential genes identified; however, the 3 previously reported genes were not among them. The lower number of genes identified in the UK cohort could be due to population diversity. Environmental effects may also play a role in the more uniform results in the Icelandic study, but further work will be needed to determine what these could be.

Despite the quiescent nature of the patient’s disease in our cohort, we were still able to identify a large number of transcriptomic changes, particularly in the rectum. This may represent a specific colonic response to inflammation or its resolution. One of the major processes identified as overexpressed in UC rectal tissue was the UPR, which has been previously shown to play a vital role in intestinal homeostasis.^46^ Dysregulation in UPR has been described in both UC and Crohn’s disease.^47^ Through the use of transgenic animal models, the functional consequence of an alteration in the UPR response and the development of gastrointestinal inflammation has been demonstrated.^43, 48^ Loss or mutations in effectors and regulators of the UPR have been shown to increase the host’s susceptibility to the development of experimental colitis.^49, 50^ Reduced numbers of genes were found to be altered in the ascending colon compared with HCs. This may be as a consequence of reduced episodes of inflammation or, in the majority of cases, no inflammation. A paucity of genes relating to UPR within the ascending colon suggests that these alterations in cellular stress could be a consequence of inflammation specifically and may not represent a “pre-inflammatory” change.

Exome sequencing identified 48 possibly damaging genetic variants with a CADD score >20 that were enriched in the UC patients. Several of the mutations were shown to have a potential influence on rectal gene expression. *SLC26A3* and coagulation factor 5 (*F5*) have both previously been identified as risk loci in IBD GWAS studies.^2, 51^*SLC26A3*, also known as the downregulated in adenoma (*DRA*) gene, encodes a protein essential for intestinal chloride absorption and is a transmembrane glycoprotein localized to the apical membrane of the columnar epithelium. Mutations in *SLC26A3* that result in loss of function have been associated with congenital chloride diarrhea.^52^ A number of SNPs have been identified in *SLC26A3* associated with either UC or IBD in both East Asian and European populations.^51, 53^

The C307W mutation in *SLC26A3* that we identified in our Icelandic UC cohort seemed to have an influence on the expression of DUOX2 and DUOXA2 in the rectum of patients. DUOX2 and DUOXA2 genes encode glycoproteins, which form a heterodimer and are members of the NADPH oxidase family. DUOX proteins are widely expressed in the apical component of the colonic epithelium and provide a mechanism of bacterial and viral killing.^54^ DUOX2 and its maturation and localization factor DUOXA2 have previously been shown to be key in the production of hydrogen peroxide within the colon and to be upregulated in active colitis and colonic cancer.^55^ They are considered to have an important role in the maintenance of the mucosal barrier and the ability to protect the host from pathogens.^56^ Recent work has identified mutations in DUOX2 in patients with IBD.^57, 58^ Further studies are needed to determine if the expression of the C307W *SLC26A3* mutation alters the DUOX2/DUOXA2 complex and has any impact on the colonic tissue or microbiota.

In addition to *SLC26A3*, the TPMT mutation A154T/Y240C also seems to have influenced rectal gene expression. Individuals who carry the A154T/Y240C variant had reduced expression of SPOP, an E3 ubiquitin ligase adaptor protein. It has most widely been described in the regulation of epithelial cell proliferation within several different cancer models including prostate, lung, ovarian, and renal cell carcinoma.^59^ SPOP is downregulated in colorectal cancers, and inactivation promotes metastasis, suggesting a role as a tumor suppressor.^60^ The exact physiological function of TPMT has yet to be elucidated; however, it is a key enzyme in the metabolism of immunosuppressive thiopurine analogues commonly used in the treatment of UC. The 2 TMPT mutations that were identified in the Icelandic UC cohort are in 100% LD and have previously been reported and given the abbreviation TMPT*3A (460G>A, rs1800460, A154T; 719A>G, rs1142345, Y240C). TMPT metabolizes thiopurine drugs, with S-adenosyl-L-methionine as the S-methyl donor and S-adenosyl-L-homocysteine as a byproduct. TMPT*3A has been shown to result in a loss of enzyme function leading to build-up of high levels of toxic metabolites from thiopurine analogues, which cause bone marrow suppression and hepatotoxicity. Genetic polymorphisms that affect enzymatic activity result in thiopurine S-methyltransferase deficiency.^37^ The increased prevalence of the TMPT variant within the Icelandic population and potentially the UC patients within this group has direct clinical relevance due to the use of thiopurine analogues as immunomodulators in this disease. Twenty percent of patients experiencing a side effect related to bone marrow suppression and leukopenia have a TPMT mutation.^61^ Screening the UC population before initiation of thiopurine therapy may be advantageous. Interestingly, in a recent study from Iceland, azathioprine was found to be the hepatotoxic drug associated with the highest risk of hepatotoxicity, affecting approximately 1 out of 130 patients treated.^62^ Very little is known about the true biological role of the TMPT, and whether it contributes to the pathogenesis of UC is also currently unclear.

In conclusion, our data provide the first paired exomic and transcriptomic data comparing patients with clinically quiescent UC and HC subjects. Transcriptomic analysis confirmed an elevation in cell cycle activity and an underlying UPR within the colonic tissue of UC patients. We were able to identify a number of predicted damaging coding variants that were present at frequencies higher than would be expected in the general Icelandic and non-Finnish European populations. Further studies including replication cohorts will be needed to determine the relevance of the variants identified to UC. In addition, it would be highly informative to determine the microbiota of the Icelandic population and UC patients. This and other possible environmental factors may help to uncover possible explanations for the increased prevalence of this disease within this population. Importantly, our findings provide evidence to support the theory that rare and damaging mutations can also have an influence on other genes within colonic tissue. Our data provide further evidence of the importance of combined multi-omics in selective populations to appreciate the impact of genomic variants on human diseases.

## SUPPLEMENTARY DATA

Supplementary data are available at *Inflammatory Bowel Diseases* online.

## Supplementary Material

Supplementary Table 1

Supplementary Table 2

Supplementary Table 3

Supplementary Table 4

Supplementary Figure 1

Supplementary Figure 2

Supplementary Data

## Notes

Supported by: We would like to thank the Medical Research Council (MR/L000261/1), which provided the funding for the study.

Author contributions to the manuscript: A.W.S., E.B.S., P.J.S., and A.M.S.: study concept and design; E.S.B., J.G.J., P.J.S., and M.V.P.: acquisition of data; M.V.P., M.F., N.P., A.P.L., and A.M.S.: analysis and interpretation of data; M.V.P., M.F., and A.M.S.: drafting of the manuscript; E.S.B., A.P.L., A.W.S., and A.M.S.: critical revision of the manuscript for important intellectual content; M.F., M.V.P., and A.M.S.: statistical analysis; A.W.S. and A.M.S.: obtained funding; E.S.B. and A.M.S. technical or material support; A.W.S. and A.M.S.: study supervision.
