# Supplementary material for: Elevation in Cell Cycle and Protein Metabolism Gene Transcription in Inactive Colonic Tissue From Icelandic Patients With Ulcerative Colitis
Source: Inflamm Bowel Dis. 2018 Nov 19;25(2):317–27. doi: 10.1093/ibd/izy350 (PMC6327231; doi:10.1093/ibd/izy350)
Supplement: Supplementary Table 2 [file izy350_suppl_supplementary_table_2.docx]

| **Gene**  **Name** | **Variant** | **ID** | **Consequence**  **Amino acid** | **CADD** | **Condel** | **Minor allele frequency (MAF)** | | | |
| --- | --- | --- | --- | --- | --- | --- | --- | --- | --- |
|  |  |  |  |  |  | **EXAC**  **non-Finnish European** | **UCLex** | **Decode reference frequency** | **UC ICE**  **Chi-squ Yates correction,**  **p-value** |
| TPMT | 6_18130918_T_C | rs1142345 | missense  Y/C | 28.3 | deleterious | 0.040 | 0.035 | 0.063 | 0.138 (0.0001) |
| TPMT | 6_18139228_C_T | rs1800460 | missense  A/T | 28.4 | deleterious | 0.036 | 0.028 | 0.062 | 0.138 (0.0001) |
| SLC26A3 | 7_107427322_A_C | rs34407351 | missense  C/W | 24.6 | deleterious | 0.049 | 0.046 | 0.057 | 0.103 (0.014) |
