# Supplementary figures and images for: Elevation in Cell Cycle and Protein Metabolism Gene Transcription in Inactive Colonic Tissue From Icelandic Patients With Ulcerative Colitis
Source: Inflamm Bowel Dis. 2018 Nov 19;25(2):317–27. doi: 10.1093/ibd/izy350 (PMC6327231; doi:10.1093/ibd/izy350)

## Slide 1
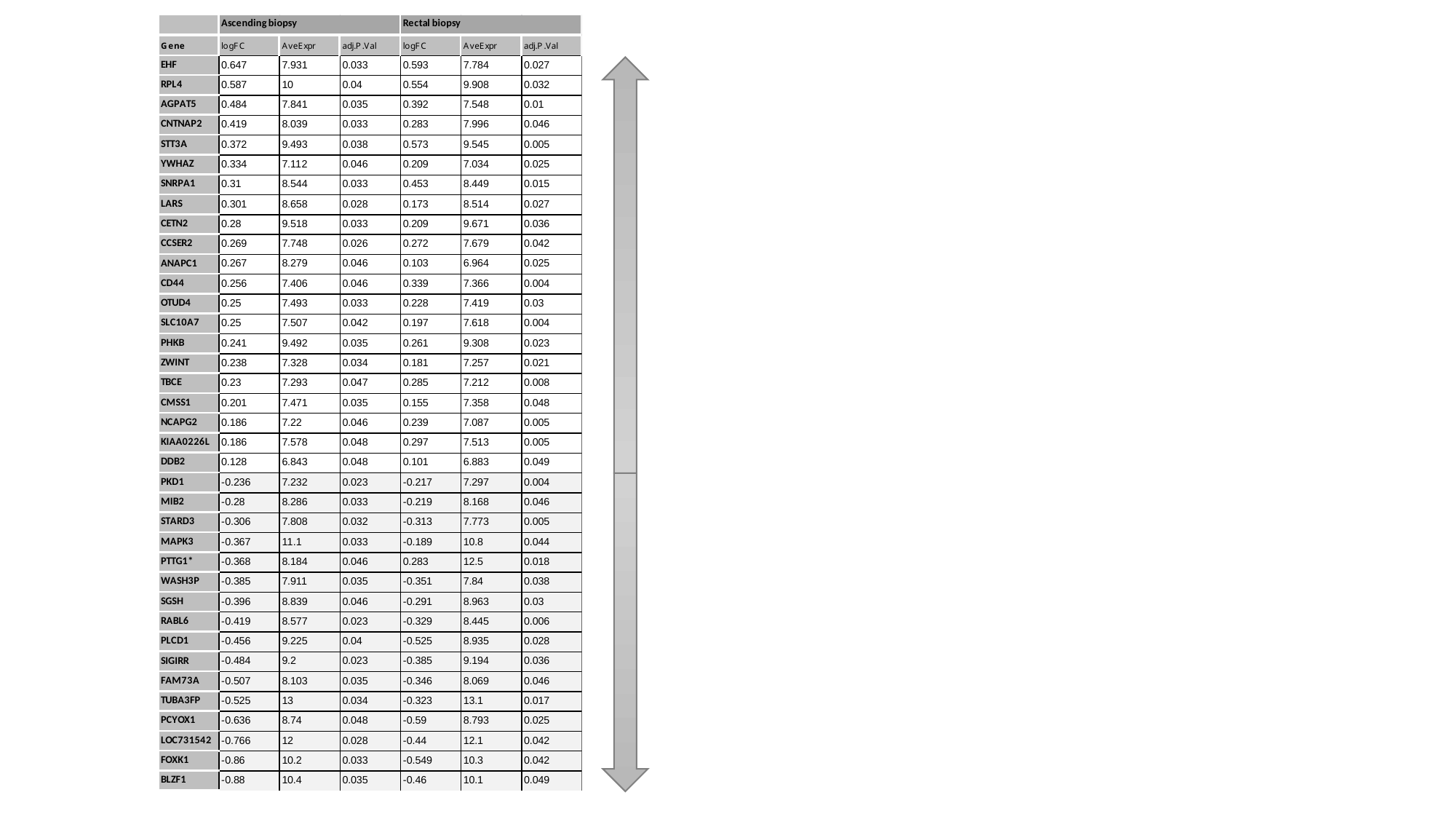

Supplement: Supplementary Table 4 [file izy350_suppl_supplementary_table_4.pptx]

## Slide 1
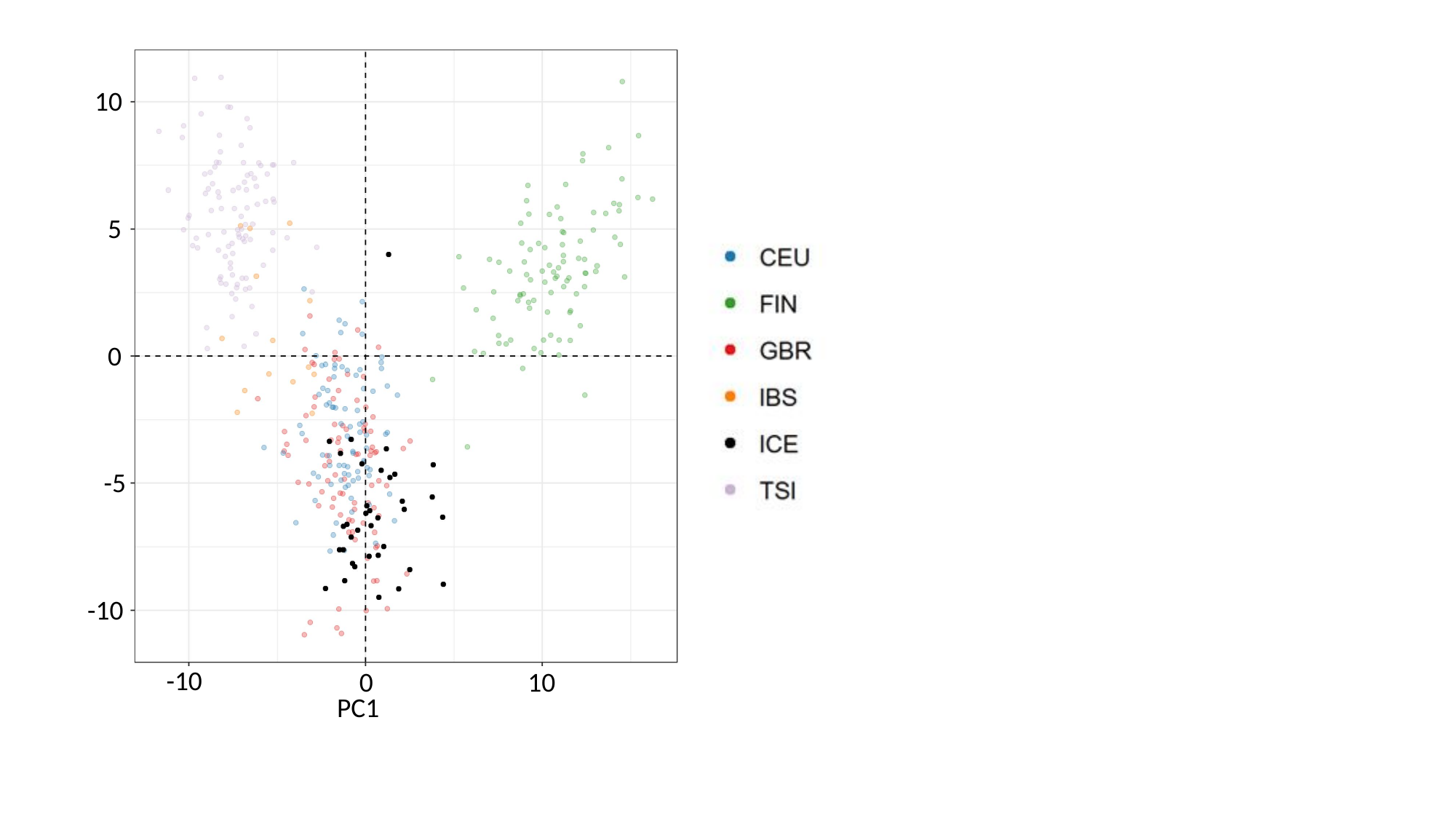

10
5
0
-5
-10
-10
0
10
PC1

Supplement: Supplementary Figure 1 [file izy350_suppl_supplementary_figure_1.pptx]

## Slide 1
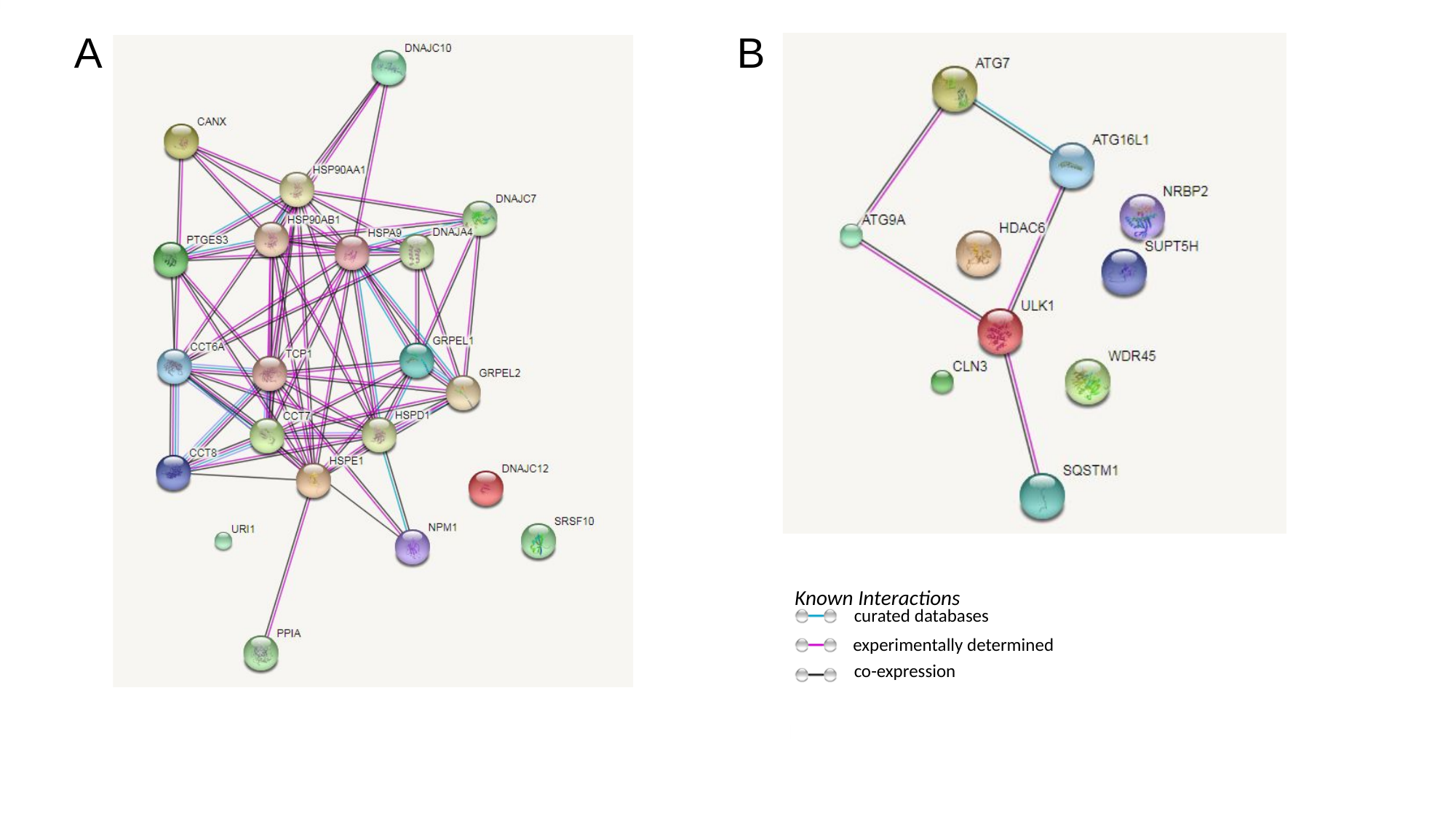

A
Known Interactions
co-expression
curated databases
experimentally determined
B

Supplement: Supplementary Figure 2 [file izy350_suppl_supplementary_figure_2.pptx]
