# Supplementary material for: Elevation in Cell Cycle and Protein Metabolism Gene Transcription in Inactive Colonic Tissue From Icelandic Patients With Ulcerative Colitis
Source: Inflamm Bowel Dis. 2018 Nov 19;25(2):317–27. doi: 10.1093/ibd/izy350 (PMC6327231; doi:10.1093/ibd/izy350)
Supplement: Supplementary Data [file izy350_suppl_supplementary_data.docx]

Supplementary data

**Table 1. Potentially damaging variants based on filtering.** 238 variants were identified, 48 have a CADD score greater than 20. Basic gene data, allele frequencies from GnomAD, our cohort and Decode data with fishers exact test and outcomes of predictive damaging variant algorithms.

**Table 2. TPMT and SLC26A3 variant.** Enrichment of TPMT variants in the UC cohort compared to non-Finnish European populations in gnomAD and the general Icelandic population (Decode data) (Fisher’s Exact Test p<0.01 was deemed significant after correction for multiple testing).

**Table 3. Differential gene expression in the rectum.** Spreadsheet including differential analysis comparing rectal gene expression in UC and HC subjects. Includes Illumina PROBE_ID, log fold change, Average Expression, t test, P.Value, adj.P.Val, gene symbol ID.

**Table 4. The overlap in differentially expressed genes for ascending and rectal pinch biopsies.** Listed in order of fold change in the ascending biopsy positive being up-regulated and negative being down-regulated in UC patients (adj p<0.05). Only one gene PTTG1* did not follow the same direction of log fold change in the rectum and ascending colon.

**Figure 1. Principal Components Analysis (PCA) generated using the exome sequence data of samples in this study and 1000 Genomes Project European populations:**  Icelandic samples from this study (ICE) and 1000 Genomes samples from the following populations - Utah Residents with Northern and Western European Ancestry (CEU), Finnish in Finland (FIN), British in England and Scotland (GBR), Iberian population in Spain (IBS) and Toscani in Italy (TSI) are represented. The first two principal components explain 0.84% and 0.49% of the overall variability.

**Figure 2. String database analysis.** Genes differentially expressed within the rectum were analysed using superpathways described by STRING. (A) 22 upregulated genes related to the UPR pathway. (B) 10 down regulated genes relating to macroautophagy were identified in the differentially expressed gene list.
